# Cross-reactivity between dengue virus and SARS-CoV-2 antibodies: Confirmation study using specimens from dengue-infected patients before the COVID-19 pandemic

**DOI:** 10.1016/j.heliyon.2024.e39099

**Published:** 2024-10-18

**Authors:** Siti Churrotin, Ilham Harlan Amarullah, Anisa Lailatul Fitria, Siti Qamariyah Khairunisa, Laura Navika Yamani, Masanori Kameoka, Novi Anggraeni, Robby Nurhariansyah, Dominicus Husada, Citrawati Dyah Kencono Wungu

**Affiliations:** a Institute of Tropical Disease, Universitas Airlangga, Surabaya, 60115, East Java, Indonesia; b Indonesia-Japan Collaborative Research Center for Emerging and Re-Emerging Infectious Diseases, Institute of Tropical Disease, Universitas Airlangga, Surabaya, 60115, East Java, Indonesia; c Master of Immunology Program, Postgraduate School of Universitas Airlangga, Surabaya, 60286, East Java, Indonesia; d Department of Nutrition, Faculty of Public Health, Universitas Airlangga, Surabaya, 60115, East Java, Indonesia; e Research Center on Global Emerging and Re-emerging Infectious Diseases, Institute of Tropical Disease, Universitas Airlangga, Surabaya, 60115, East Java, Indonesia; f Department of Epidemiology, Biostatistic, Population Studies and Health Promotion, Faculty of Public Health, Universitas Airlangga, Surabaya, 60115, East Java, Indonesia; g Department of Public Health, Kobe University Graduate School of Health Sciences, 7-10-2 Tomogaoka, Suma-ku, Kobe, Hyogo, 654-0142, Japan; h Ngudia Husada Madura Midwifery Academy, Bangkalan, Madura, 69116, Indonesia; i Department of Child Health, Universitas Airlangga Hospital, Surabaya, 60115, Indonesia; j Department of Child Health, Faculty of Medicine, Universitas Airlangga/Dr Soetomo General Academic Hospital, Surabaya, 60132, Indonesia; k Department of Physiology and Medical Biochemistry, Faculty of Medicine, Universitas Airlangga, Surabaya, 60132, East Java, Indonesia

**Keywords:** *Dengue*, *SARS-CoV-2*, *Cross-reactivity*, *Antibody*, *COVID-19*

## Abstract

**Background:**

The simultaneous occurrence of the COVID-19 pandemic and a dengue outbreak has posed significant challenges for governments and medical personnel in dengue-endemic countries like Indonesia. Several studies in dengue-endemic countries have reported cases of misdiagnosis between COVID-19 and dengue. Therefore, it is crucial to evaluate the potential cross-reactivity between SARS-CoV-2 antibodies and dengue.

**Methods:**

This study aimed to confirm the serological cross-reaction between dengue virus and SARS-CoV-2 in Surabaya, East Java, which is a highly dengue-endemic city in Indonesia. In total, 238 serum samples with confirmed dengue that were collected before the emergence of COVID-19 were tested to detect the presence of reacting IgG and IgM antibodies (Abs) against SARS-CoV-2 via a rapid detection test (RDT) and enzyme-linked immunosorbent assay (ELISA). Samples from patients with dengue infection collected during the pandemic, from healthy volunteers predating the pandemic, and from patients with COVID-19 were used for comparison.

**Results and conclusion:**

Few (6.7 %) of the pre-COVID-19 dengue Ab-positive serum samples showed reactive on SARS-CoV-2 in the RDT, with significantly lower IgG and IgM levels detected in ELISA compared with the dengue samples collected during the pandemic and the COVID-19 samples (*P* < 0.005). A comparable anti-SARS-CoV-2 IgG concentration was observed in the pre-COVID-19 dengue samples and healthy volunteers (*P* = 0.56), which also indicated other possibilities. In conclusion, our results suggested a low risk of cross-reactivity between dengue virus and SARS-CoV-2. However, they highlighted the need for caution when using and interpreting data obtained stemming from serological methods, to prevent false-positive results. Further studies are needed to evaluate the cross-reactivity between dengue virus, SARS-CoV-2, and other common human pathogens, as well as its effect on the serosurveys, treatment of these diseases, or vaccine efficacy.

## 1 Introduction

Since its initial discovery in Wuhan in November 2019, the human severe acute respiratory syndrome coronavirus 2 (SARS-CoV-2) had spread globally, with 624,235,272 confirmed cases of infection and 6,555,270 deaths as per October 18, 2022 [1]. Indonesia reported the first case of the coronavirus diseases 2019 (COVID-19) in early March 2020, and 6,472,664 confirmed cases have been recorded as per October 25, 2022, resulting in a mortality rate of 2.5 % [2].

The emergence of the COVID-19 pandemic in the midst of a simultaneous dengue outbreak has become a new challenge in dengue-endemic countries. The fact that COVID-19 can camouflage during the early stages of infection followed by the long incubation period opens up opportunities for an early misdiagnosis of dengue infection [3]. Several countries in the Southeast Asian region have reported cases of COVID-19 disguised as dengue infection. Thailand recorded the first death of a patient with COVID-19 who was misdiagnosed as dengue infection because of the presence of a red rash, typical dengue symptom [4]. Yan G. et al. also stated that there were similarities in clinical and laboratory characteristics between dengue infection and COVID-19 in two cases diagnosed in Singapore. At the initial diagnosis, both patients showed typical symptoms of dengue infection and positive dengue IgM, However further examination confirmed as SARS-CoV-2 infection [5].

We have been conducting dengue virus surveillance in Surabaya, East Java, which is the second largest city in Indonesia, from 2008 up until recently. The results of our study showed an association between an increase in the number of cases and disease severity and changes in serotype and genotype of dengue virus [[6], [7], [8]]. During the COVID-19 pandemic, Surabaya became COVID-19 red zone showing highest number of cases in East Java. In response to the dynamics that ensued, we conducted a preliminary study of the potential cross-reactivity between SARS-CoV-2 and dengue antibodies in specimens of COVID-19 patients. The results indicated that 4 out of 123 samples were reactive to dengue IgG but were not positive for the nonstructural 1 (NS1) antigen test or real-time polymerase chain reaction (RT-PCR) [9]. Nevertheless, the presence of dengue IgG in individuals living in endemic areas cannot be ignored.

Some studies have suggested that individuals with previous exposure to dengue may have higher levels of cross-reactive antibodies to SARS-CoV-2 [10,11]. Interestingly, there is report indicating that the severity and transmission of SARS-CoV-2 were low in dengue endemic countries [12]. However, it was not clear whether the pre-exposure to dengue virus provided some extent of protection against COVID-19 severity or whether misdiagnosed cases of COVID-19 caused the misreporting of data. Several studies confirmed the potential of cross-reactivity between dengue virus and SARS-CoV-2 antibodies [[13], [14], [15]]. In contrast, a study conducted in the United States and Puerto Rico suggested that the serological assays of SARS-CoV-2 are not affected by dengue virus and vice versa [15]. A study of the pre-COVID-19 samples from a dengue-infected patient reported a false positive band in the SARS-CoV-2 IgG and IgM rapid detection tests, with a positivity rate of 38.5 % [16]. Docking studies also revealed that dengue virus antibody can bind to SARS-CoV-2 receptor-binding domain (and the reverse scenario) [16]. A computational prediction indicate that dengue antibodies were capable of intercepting eight key RBD interactions that are crucial for binding to angiotensin converting enzyme 2 (ACE2) receptors, which theoretically could camouflage as the SARS-CoV-2 RBD and block its interaction with host cell receptors, thereby preventing viral entry [17].

Considering the importance of addressing this cross-reactivity issue, here we conducted a cross-sectional study of the cross-reactivity between dengue virus and SARS-CoV-2 antibody. A larger size of pre-COVID-19 dengue-confirmed samples was employed in our observation to ensure the result validity.

## 2 Data and methods

### 2.1 Data sources

#### 2.1.1 Test specimens

Three serum panels were used in this study including dengue positive predating the COVID-19 pandemic (n = 238), dengue positive during the COVID-19 pandemic (n = 12), healthy controls (n = 38), and COVID-19 positive plasma (n = 20; as a comparative positive control).

**Fig. 1:**
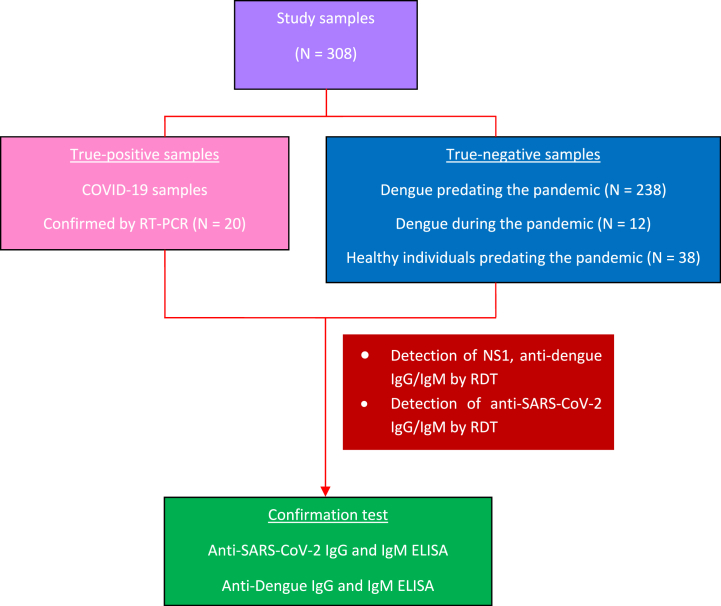
Workflow of this research.

Dengue positive samples were obtained from clinically diagnosed patients with dengue fever or dengue hemorrhagic fever during the acute phase at 1–5 days after symptom onset and showed positive result in at least one of the tests including NS1, IgG, IgM, and RT-PCR serotyping. Dengue samples predating the COVID-19 pandemic were collected in 2014–2019 in Surabaya. Dengue samples during the COVID-19 pandemic were collected in April–May 2021 and October 2021–May 2022 from patients in Universitas Airlangga hospital. Those patients had not received any COVID-19 vaccination, had no history of COVID-19 (interview data), and were declared as being negative for COVID-19 on the enrollment day of hospitalization using swab and real time PCR method. The confirmation of a negative COVID-19 status was also strengthened by continuous monitoring of clinical and laboratory observations over the hospitalization period to ensure there was no SARS-CoV-2 coinfection. Healthy controls were defined as dengue negative confirmed by NS1, and IgG/IgM test which were collected in August 2014. The positive controls were obtained from individuals who were proved COVID-19 positive by real time PCR test at the Institute of Tropical Disease, Universitas Airlangga. Plasma was collected 2 weeks after disease onset according to the self-reported information provided by patients in the consent form. Samples were collected through consecutive method with an exclusion criteria for autoimmune indivuals and chronic steroid users. All samples were kept at −80 °C prior to usage. The workflow of the present study is presented in Fig. 1.

#### 2.1.2 Dengue NS1 antigen and IgG/IgM antibodies rapid detection test (RDT)

The detection of NS1 antigens and the dengue-virus-specific IgG,/IgM was carried out using the Standard Diagnostics-Bioline Dengue Duo rapid test kit (SD Biosensor, Suwon-si, Korea). 100 μl of sample was dropped to the sample well of the NS1 test device, while 10 μl of sample was dropped to the sample well of the IgG/IgM test device with addition of 90 μl of assay diluent. The results were read within 15–20 min. The interpretation of the test results was according to the manufacturer's instructions.The NS1 antigen kit yielded 92.4 % (95 % confidence interval [CI]: 86.1%–95.9 %) sensitivity and 98.4 % (95 % CI: 95.5%–99.5 %) specificity. For the detection of dengue IgG/IgM, the kit showed 94.2 % (95 % CI: 88.5%–97.2 %) sensitivity and 96.4 % (95 % CI: 93.0%–98.2 %) specificity.

#### 2.1.3 SARS-CoV-2 IgG/IgM antibodies rapid detection test (RDT)

SARS-CoV-2 antibodies of the samples were examined using an anti-SARS-CoV-2 IgM/IgG kit (Vazyme Medical Technology, Nanjing, China). 20 μl of sample and 60 μl of assay diluent were loaded to the sample well of the IgG/IgM test device. The results were observed within 10 min. The analysis of the test results was according to the manufacturer's instructions. The kit had a sensitivity of 91.54 % (95 % CI: 86.87%–94.65 %) and a specificity of 97.02 % (95 % CI: 94.74%–98.33 %).

#### 2.1.4 SARS-CoV-2 antibodies enzyme-linked immunosorbent assay (ELISA)

For further validation, the samples were also tested using SARS-CoV-2 anti-RBD IgG and IgM detection ELISA kits, EZRBDM-110K and EZRBDG-120K, respectively (EMD Millipore Corporation, Billerica, USA). These kits detect antibodies in the serum and plasma. Positive controls, negative controls and an RBD monoclonal IgG/IgM standard were used in each plate. The ELISA was performed as per the manufacturer's protocols using 1:100 diluted serum samples. The optical density (OD) was read at 450 nm wavelength using a BioRad iMarck microplate reader Seri 17539. The lower limit of quantification of human SARS-CoV-2 anti-RBD IgG and IgM was 1.56 ng/ml and 0.78 ng/ml, respectively.

#### 2.1.5 Dengue virus antibodies enzyme-linked immunosorbent assay (ELISA)

As a validation to assessment of dengue virus antibodies by RDT, we conducted a qualitative ELISA for samples showing cross-reactivity in dengue predating pandemic and dengue during pandemic, all healthy and COVID-19 controls. Dengue virus capture IgG and IgM ELISA kits, MBS494059 and MBS580101, respectively (MyBiosource, San Diego, USA) were used to detect antibodies in the serum and plasma. The calibrator, positive and negative controls were used in each plate. The test samples were diluted 21 times in sample diluent in the assays. All ELISA steps were performed at room temperature. The OD was read at 450 nm wavelength using BioRad iMarck microplate reader Seri 17539. Samples were considered positive according to the standard protocols of the manufacturer; for dengue IgG (Standard units <9 is negative; 9–11 is equivocal; and >11 is positive) and for dengue IgM (Antibody index <0.9 is negative; and >9 is positive).

### 2.2 Data processing

#### 2.2.1 Statistical analysis

Statistical analyses were performed using GraphPad Prism 9 (GraphPad Software, Inc., San Diego, California, US). The median (IQR) and percentage frequency were used to analyze the data. A normality test was performed on the numerical data using the Shapiro–Wilk test. Data were analyzed using the Kruskal–Wallis test, followed by the Mann–Whitney *U* test for significant variables. Significance was set at *P* < 0.05. For comparing SARS-CoV-2 antibodies level between three groups (predating pandemic, during pandemic, and COVID-19 patients), Kruskal Wallis test was used, followed by Mann Whitney post hoc test. To analyze SARS-CoV-2 antibodies level between positive-negative NS1, dengue IgG and IgM, we used Mann Whitney test.

## 3 Results

### 3.1 Antibodies assessment using RDT

Dengue antibodies detection using RDT (Table 1) was first carried out showing that the majority of dengue patients were IgG positive as indicator of secondary infection. In dengue predating pandemic, most of the samples (47.9 %) were negative for NS1 antigen with positive dengue IgG. Both IgG and IgM positive were found the highest (58.3 %) in dengue during pandemic samples with NS1 negative. No positive NS1 antigen and dengue antibodies were detected in healthy volunteer and COVID-19 patients using RDT.

**Table 1:** Profile of dengue antibodies among test groups assessed by RDT.

Test Groups	NS1 antigen	Dengue antibodies			
		IgG+/IgM-	IgG-/IgM+	IgG+/IgM+	IgG-/IgM-
Dengue predating pandemic	Positive	6.7 % (16/238)	2.1 % (5/238)	4.6 % (11/238)	0 % (0/238)
Dengue during pandemic		0 % (0/12)	16.7 % (2/12)	16.7 % (2/12)	0 % (0/12)
Healthy Volunteer predating pandemic		0 % (0/38)	0 % (0/38)	0 % (0/38)	0 % (0/38)
COVID-19 patients		0 % (0/20)	0 % (0/20)	0 % (0/20)	0 % (0/20)
Dengue predating pandemic	Negative	47.9 % (130/238)	13.9 % (33/238)	24.8 % (70/238)	0 % (0/238)
Dengue during pandemic		16.7 % (2/12)	0 % (0/12)	58.3 % (8/12)	0 % (0/12)
Healthy Volunteer predating pandemic		0 % (0/38)	0 % (0/38)	0 % (0/38)	0 % (0/38)
COVID-19 patients		0 % (0/20)	0 % (0/20)	0 % (0/20)	0 % (0/20)

**Fig. 2:**
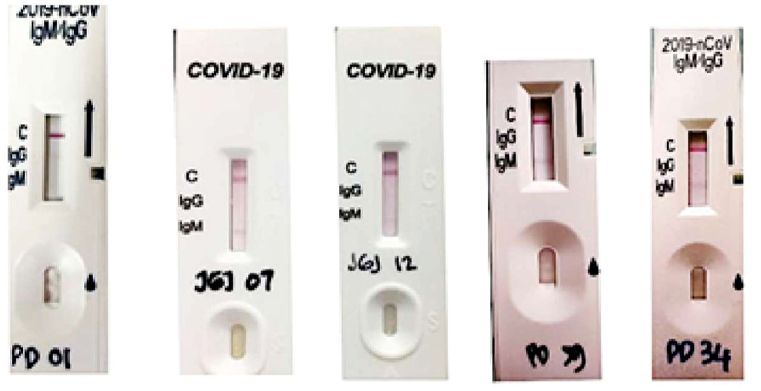
Results of the rapid detection test for SARS-CoV-2 antibody reactivity for dengue samples collected before the COVID-19 pandemic.

Subsequently, samples were also subjected to SARS-CoV-2 antibodies RDT. Out of 238 dengue collected before the pandemic, 6.7 % (16/238) samples were reactive to SARS-CoV-2 antibodies, in detail 2.1 % (5/238) IgG+/IgM-, 4.2 % (10/238) IgG-/IgM+, 0.4 % (1/238) IgG+/IgM+. Dengue during pandemic samples were reactive only on IgG 75 % (9/12). No dengue antibodies were detected in healthy predating pandemic samples. All of COVID-19 samples were positive on both SARS-CoV-2 IgG and IgM antibodies (Table 2). Most of the positive results from dengue samples showed a faint band on SARS-CoV-2 RDT, indicating that the concentration of the antibody was not high. (Fig. 2).

**Table 2:** Assessment of cross-reactivity with SARS-CoV-2 antibodies using RDT.

Test Groups	SARS-CoV-2 antibodies			
	IgG+/IgM-	IgG-/IgM+	IgG+/IgM+	IgG-/IgM-
Dengue predating pandemic	2.1 % (5/238)	4.2 % (10/238)	0.4 % (1//238)	0 % (0/238)
Dengue during pandemic	75 % (9/12)	0 % (0/12)	0 % (0/12)	0 % (0/12)
Healthy Volunteer predating pandemic	0 % (0/38)	0 % (2/38)	0 % (0/38)	0 % (0/38)
COVID-19 patients	0 % (0/20)	0 % (0/20)	100 % (20/20)	0 % (0/20)

### 3.2 Antibodies assessment using ELISA

To confirm the results obtained by RDT, we assessed the SARS-CoV-2 antibodies using ELISA, which detected antibodies directed against the SARS-CoV-2 spike (RBD) protein, as displayed on Table 3. Among the RDT SARS-CoV-2 positive in dengue predating pandemic samples, we observed 62.5 % (10/16) IgG-/IgM+ and 37.5(6/16) IgG+/IgM+. Unlike RDT data, there was no positive IgG+/IgM-found. IgM in dengue during pandemic samples, which previously showed negative in RDT, were reactive in ELISA. Likewise, the healthy volunteer samples gave different results indicating 63.2 % (24/38) IgG-/IgM+ and 36.8 % (14/38) IgG+/IgM+.

**Table 3:** Assessment of cross-reactivity with SARS-CoV-2 antibodies using ELISA.

Test Groups	SARS-CoV-2 antibodies			
	IgG+/IgM-	IgG-/IgM+	IgG+/IgM+	IgG-/IgM-
Dengue predating pandemic	0 % (0/16)	62.5 % (10/16)	37.5 % (6/16)	0 % (0/16)
Dengue during pandemic	0 % (0/9)	0 % (0/9)	100 % (9/9)	0 % (0/9)
Healthy volunteer predating pandemic	0 % (0/38)	63.2 % (24/38)	36.8 % (14/38)	0 % (0/38)
COVID-19 patients	0 % (0/20)	5 % (1/20)	95 % (19/20)	0 % (0/20)

The qualitative ELISA was employed to confirm the presence of dengue antibodies in dengue predating pandemic that reactive to SARS-CoV-2 antibodies in RDT, during pandemic samples that reactive to SARS-CoV-2 antibodies in RDT, all the healthy and COVID-19 controls (Table 4).

**Table 4:** Results of qualitative dengue antibodies measurement with ELISA.

Test Groups	Dengue antibodies			
	IgG+/IgM-	IgG-/IgM+	IgG+/IgM+	IgG-/IgM-
Dengue predating pandemic	25 % (4/16)	25 % (4/16)	6.25 % (1/16)	43.75 % (7/16)
Dengue during pandemic	33.3 % (3/9)	33.3 % (3/9)	50 % (6/9)	0 % (0/9)
Healthy volunteer predating pandemic	10.5 % (4/38)	5.3 % (2/38)	0 % (0/38)	84.2 % (32/38)
COVID-19 patients	20 % (4/20)	0 % (0/20)	0 % (0/20)	0 % (0/20)

Among dengue predating pandemic that reactive to SARS-CoV-2 antibodies (N = 16), 56.25 % of the samples exhibited positivity for dengue IgG and/or IgM antibodies by ELISA. However, the other 43.75 % of the samples showed negative dengue antibodies, a condition that was not observed in the assessment by dengue antibodies RDT. Among dengue during pandemic that reactive to SARS-CoV-2 antibodies (N = 9), 50 % of samples were positive for both IgG and IgM dengue antibodies and 33.3 % for either only IgG or IgM. This group showed a relative similar positivity rate on dengue antibodies detected by RDT and ELISA. The healthy predating pandemic and COVID-19 showed different positivity results from those obtained with RDT. Dengue antibodies were detected in some samples of these groups. Out of 38 healthy controls, 10.5 % samples exhibited dengue IgG and 5.3 % exhibited IgM antibody. While, dengue IgG antibody was detected in 20 % of COVID-19 samples.

### 3.3 Analysis of cross-reactivity between dengue and SARS-CoV-2 antibodies

**Fig. 3:**
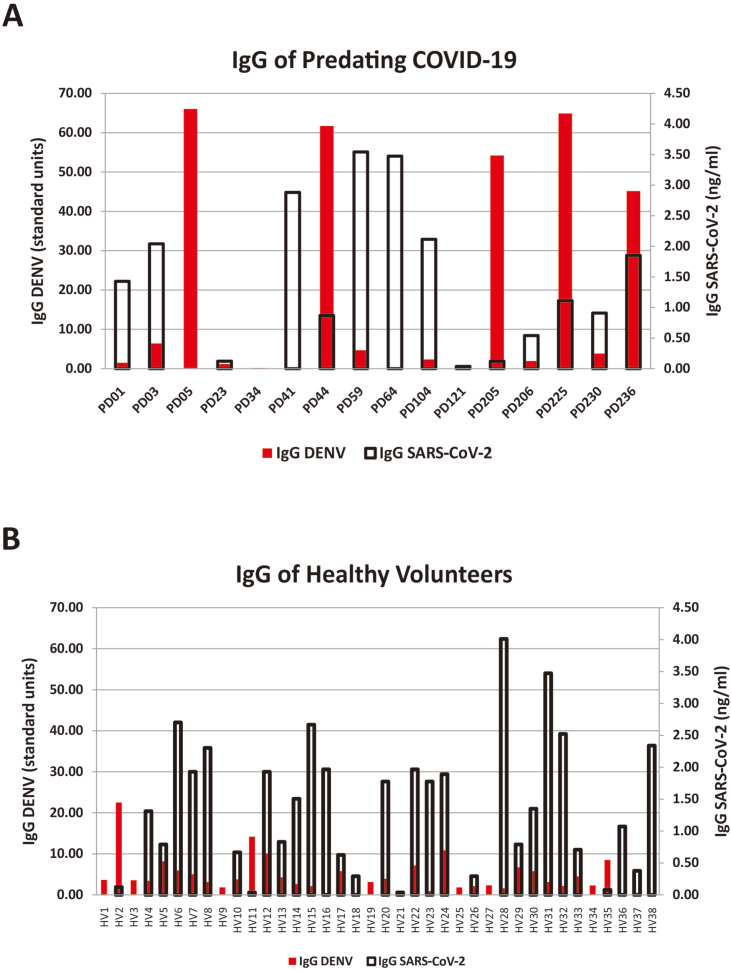
Comparison of IgG level between ELISA Dengue vs SARS-CoV-2 in each sample of: (A) predating COVID-19; (B) healthy volunteers.

**Fig. 4:**
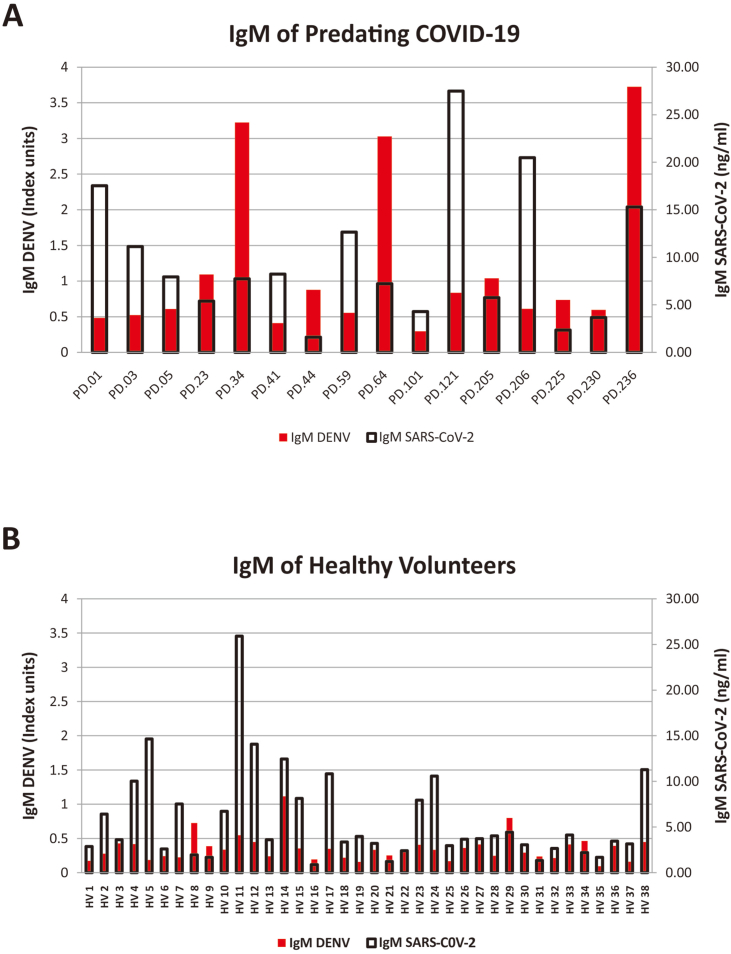
Comparison of IgM level between ELISA Dengue vs SARS-CoV-2 in each sample of: (A) predating COVID-19; (B) healthy volunteers.

The potential cross-reactivity between dengue virus and SARS-CoV-2 antibodies in this study was analyzed by comparing the obtained data from RDT and ELISA (Supplementary Table 1). The ELISA results of dengue predating and healthy volunteer group are depicted in the graph to compare the concentration level of IgG (Fig. 3) and IgM (Fig. 4) in the sample.

ELISA Dengue IgG show positive results for dengue predating group (Fig. 3A) in PD05, PD44, PD205, PD225, and PD236, while ELISA SARS-CoV-2 IgG positive are shown by PD03, PD41, PD59, PD64, PD104, and PD236. Only PD236 indicates positive for Dengue and SARS-CoV-2. IgG positivity in healthy volunteer group (Fig. 3B) were observed higher for SARS-CoV-2, while only four samples displayed positive Dengue IgG (HV02, HV11, HV12, and HV24). On the other hand, ELISA SARS-CoV-2 IgM indicated positive results for all samples. ELISA Dengue IgM for dengue predating (Table 4A) and healthy volunteers (Table 4B) showed five and one positive results, respectively.

### 3.4 Comparison of SARS-CoV-2 antibodies in cross-reactive samples

The results of the Kruskal–Wallis analysis revealed significant differences between the levels of IgG and IgM (as assessed using the anti-SARS-CoV-2 ELISA kit) among the groups (Table 5).

**Table 5:** Comparison of the IgG and IgM levels on anti-SARS-CoV2 ELISA in the four groups.

Groups	Median (IQR) conc. of SARS-CoV-2 ELISA IgG (ng/mL)	*P* value	Median (IQR) conc. of SARS-CoV-2 ELISA IgM (ng/mL)	*P* value
COVID-19 positive	174.4 (101.1–350.4)	<0.0001^a^	54.78 (20.33–102.4)	<0.0001^a^
Predating the pandemic	1.157 (0.53–1.95)		7.78 (4.54–14.56)	
During the pandemic	52.08 (27.76–112.8)		4.61 (2.28–6.07)	
Healthy volunteers	1.01 (0.47–1.83)		3.63 (2.64–7.94)	

a Significant at *P* < 0.05.

The post-hoc Mann–Whitney *U* test showed a significant difference between the IgG and IgM levels obtained in the SARS-CoV-2 ELISA in almost all groups. However, there was no significant difference in the levels of IgG on SARS-CoV-2 ELISA between the predating group and the healthy volunteers, or in the levels of IgM on SARS-CoV-2 ELISA between the samples collected during the pandemic and healthy individuals (Table 6 and Supplementary Fig. 1).

**Table 6:** Results of the post hoc Mann–Whitney *U* test of differences in IgG and IgM anti-SARS-CoV2 ELISA levels among the four groups.

ELISA SARS-CoV2 Antibodies	Groups	COVID-19	PD	D	Healthy
IgG	COVID-19		<0.0001^a^	0.006^a^	<0.0001^a^
	PD	<0.0001^a^		<0.0001^a^	0.564
	D	0.006^a^	<0.0001^a^		<0.0001^a^
	Healthy	<0.0001^a^	0.564	<0.0001^a^	
IgM	COVID-19		0.0002^a^	0.0003^a^	<0.0001^a^
	PD	0.0002^a^		0.022^a^	0.013^a^
	D	0.0003^a^	0.022^a^		0.676
	Healthy	<0.0001^a^	0.013^a^	0.676	

a Significant at *P* < 0.05. PD = dengue sample predating the pandemic; D = dengue sample during the pandemic.

## 4 Discussion

To the best of our knowledge, this was the first Indonesian study that evaluated the presence of SARS-CoV-2 IgM and/or IgG antibodies in a high number of dengue-confirmed samples predating the COVID-19 pandemic-with the primary objective of confirming the cross-reactivity. DENV and SARS-CoV-2 antibodies evaluated with the serological methods described in this study showed reactivity with different rate in each sample group namely dengue predating pandemic, dengue during pandemic, healthy volunteers predating pandemic and COVID-19 patients.

We identified a low false-positive rate 6.7 % (16/238) among dengue predating pandemic samples on SARS-CoV-2 IgG/IgM RDT. Using a smaller number of sample, another study reported that 3.3 % of the dengue samples in Indonesia were cross-reactive with antibodies for anti-SARS-CoV-2 [18]. Our finding is consistent with a study conducted by Italian researchers that employed the same scenario, who obtained a serological cross-reactivity of only 2.3 % between dengue and COVID-19 in 44 samples collected from Italian travelers returning from dengue-endemic countries before SARS-CoV-2 emerged [19].

The obtained result of IgG/IgM RDT analysis displays similarity in more than half amount of Dengue predating pandemic samples. Among 12 Dengue IgG positive, 4 samples were reactive to SARS-CoV-2 IgG. Dengue IgM positive samples (n = 8) also showed 5 reactive SARS-CoV-2 IgM (Supplementary Table 1). This might be an indication of cross reactivity of the dengue antibodies. Regarding RDT analysis, we choose to rule out the possibilities of cross reactivity with other common coronavirus family since the manual guide of the vazyme stated that the RDT will not cross-react with many listed viruses including common coronavirus 229E, HKU-1, OC43, and NL63. However, dengue does not belong to those listed viruses. We chose vazyme since it is one of the top 10 brands widely used in Indonesia and it belongs to the recommended brands to be used by Indonesian COVID-19 Response Acceleration Task Force [20]. A study to examine vazyme indicated this RDT did not cross react with common coronavirus 229E, HKU-1, OC43, and NL63 [21]. Rather than the different number of tested samples, we believe that the cross-reactivity rate on the rapid diagnostic test was affected by the kit characteristics such as the base of the protein target used, the sensitivity and specificity. A comparison study of three SARS-CoV-2 antibodies RDTs including Hangzhou Alltest, SD Biosensor, and Vazyme show that the previous two, which use N protein as the antigen, indicated better performance [22].

Serum samples of dengue during pandemic show 75 % were positive for SARS-CoV-2 IgG only, despite being declared as COVID-19 negative. Rather than cross-reactivity, this result could be an indication of asymptomatic SARS-CoV-2 infection prior to their hospitalization period. Indeed, the patients’ enrollment to hospital employed strict screening and continuous monitoring to ensure no SARS-CoV-2 infection was done. Nevertheless, the screening was done by swab and real time PCR method. Furthermore, the history of COVID-19 was confirmed only by interview. Meanwhile. COVID-19 has been widely reported, asymptomatic infections are not new. An RDT-based serosurvey performed in East Java, Indonesia, revealed an IgG prevalence of 14.6 % in non-COVID-19 patients during the pandemic [23]. In addition, a study performed in the early days of the pandemic reported that the percentage of asymptomatic COVID-19 could reach 81 % [24]. Moreover, a systematic review and meta-analysis found a global asymptomatic SARS-CoV-2 infection rate of approximately 40%–45 % [25].

After the serological test using RDT, a quantitative ELISA anti-RBD IgM/IgG test and ELISA anti DENV IgG/IgM was performed in this study. ELISA was reported to have a higher accuracy for COVID-19 serologic diagnosis compared with the RDT, with a significantly improved sensitivity, whereas it combined IgG and IgM in one test [26,27]; therefore, ELISA was used to confirm the serostatus and assess the level of SARS-CoV-2 antibodies. Furthermore, Shurrab et al. validated that the analysis results obtained using ELISA were consistent with those obtained using chemiluminescence immunoassay (CLIA). Their findings corroborated our own, indicating low cross-reactivity in rapid detection test was confirmed by both ELISA and CLIA [28].

The SARS-CoV-2 IgG antibodies were not only detected in the confirmed COVID-19 samples, but also in the other sample groups with a wide range of concentrations. On the other hand, SARS-CoV-2 IgM was detected in all samples. The cross-reactivity rate in COVID-19-predating dengue and during pandemic dengue samples was similar in the RDT and ELISA. A higher percentage of IgG reactivity was detected in healthy-volunteer samples which were in contrast with the findings of the RDT. Comparing the ELISA data, although the rate of reactivity was comparable between ELISA DENV and ELISA SARS-CoV-2 in dengue predating samples, however there is no similarity pattern of the positive samples. Only PD-236 showed identical result (Supplementary Table 1). Furthermore, comparison of ELISA and RDT for dengue predating samples also has no similarity result. One of the possible reasons behind this cross reactivity might happen not due to the IgG but other molecules of dengue virus. A study conducted by Yi-Ling Cheng et al. indicated that anti-S1-RBD cross react with dengue virus proteins including E, prM, and NS1. The strongest cross reactivity was with E protein and no cross reactivity with NS4 protein. His group also found there is similarity in the epitope of SARS-CoV-2 S1-RBD and DENV E protein at the location of amino acid 343–347 and 64–69, respectively. Both of these epitopes are located on the surface area. In addition, NS1 sequence at amino acid 115–119 also shares similarity with S1-RBD 376–380 [29].

The ELISA kit for SARS-CoV-2 IgG/IgM in this study utilized anti-S1-RBD principle. Furthermore, our archived dengue positive samples were collected during acute-phase. Because dengue samples in our study group were mainly proposed for the molecular detection of viral RNA, which implies that this study did not address cross-reactivity during the convalescence phase. Based on that, there is a possibility that the serum still contains other DENV proteins molecules (E and prM) which could react to SARS-CoV-2 serological tests resulting in different pattern of positivity among samples.

Another possibility is that cross-reactivity observed in ELISA, particularly in healthy volunteer samples, might be caused by preexposure to common cold coronavirus circulating in Indonesia. We could not rule this out completely since there is no statement or information regarding product cross reactivity. According to national data from 2018, the seroprevalence of the common cold virus in Indonesia was reported to be 25 %, of which only 13.8 % cases were medically diagnosed [30]. The *Coronaviridae* family comprises seven strains that can infect humans, including MERS-CoV, SARS-CoV, SARS-CoV-2, 229E, HKU-1, OC43, and NL63. The four latter strains are distributed globally and only cause moderate symptoms in the upper respiratory tract. Therefore, they cause just the common flu and perhaps these patients would not seek medical treatment, implying that the virus would not be detected or documented well [31]. In addition, it was also reported that SARS-CoV-2–reactive CD4^+^ T cells were detected in unexposed individuals, indicating the presence of pre-existing cross-reactive T-cells in 20%–50 % of the population [32,33]. Moreover, an epitope-mapping study showed a variety of pre-existing memory CD4^+^ T cells exhibiting a comparable affinity for cross-reactivity with SARS-CoV-2 and the coronaviruses responsible for the common cold. Therefore, the considerable heterogeneity observed in COVID-19 may be partially explained by a variable T-cell memory to the coronaviruses that cause the common cold [34]. Moreover, the IgG cross-reactivity to common human coronaviruses is reportedly high, triggered by the conserved antigenic S2 domain of the spike and nucleocapsid proteins [35].

The SARS-CoV-2 IgM ELISA displayed all samples being positive including the healthy volunteer. We are aware that this result needs to be further validated. Unfortunately, due to limited resource and budget in our study, it could not be done. However, comparison on the concentration level among sample groups shows that healthy volunteers give the lowest median value 3.63 (2.64–7.94) ng/ml. The predating dengue show two-fold higher in concentration 7.78 (4.54–14.56) ng/ml while COVID-19 positive samples indicate 13-fold higher than the healthy volunteer group. Some studies reported that SARS-CoV-2 IgM was more cross-reacting with antibodies acquired from dengue and acute febrile illness on serological assays [13,15]. This might be the reason why the concentration of dengue predating and dengue during pandemic showed relatively higher value.

Another limitation of the study was the unequal sample sizes of the groups included in this study. The sample collection was challenged by the limited resources of time and budgeting. During the COVID-19 pandemic, a significant decrease in the number of patients with dengue occurred in Indonesia, and, consequently, also in the hospital where we collected the samples. Therefore, few dengue samples were available during the COVID-19 pandemic that met the criteria for inclusion as the comparative group. In turn, because of a tight grant budget, we selectively set the number of confirmed COVID-19 samples and healthy volunteer samples to be tested. However, as we used a series of qualified samples, we believe that this suboptimal sample-size conditions did not have a substantial impact on the power of confidence of this study.

The fact that we did not measure the level of the dengue antibodies was another flaw of this study. It would be more reliable to determine the level of dengue antibodies beforehand and perform a correlation study of levels of those two types of antibodies. Therefore, as a follow-up of this study, the development of a gold-standard method is necessary to clarify the serological cross-reactivity of dengue and SARS-CoV-2. Second, to rule out the impact of a preexisting cross-reactive dengue immunity and vaccine efficacy, a neutralization test of dengue samples against SARS-CoV-2 is of utmost importance. It would be also ideal to examine the samples using a kit with validated cross-reactivity testing data for other viruses.

## 5 Conclusion

In conclusion, our results suggest that there is a potential low risk of cross-reactivity between dengue and SARS-CoV-2 antibodies (IgG/IgM) in the populations of the dengue-endemic region in Indonesia. This emphasizes the importance of taking caution when using and interpreting the serological results of serological methods, especially in dengue-endemic areas, to avoid false-positive results. In addition, further research using more –specific testing and an optimum cut-off value is important to draw definite conclusion of cross reactivity issue.

## CRediT authorship contribution statement

**Siti Churrotin:** Writing – original draft, Investigation, Data curation, Conceptualization. **Ilham Harlan Amarullah:** Writing – review & editing, Project administration, Investigation. **Anisa Lailatul Fitria:** Writing – review & editing, Validation, Investigation, Data curation. **Siti Qamariyah Khairunisa:** Writing – review & editing, Investigation, Formal analysis, Data curation. **Laura Navika Yamani:** Supervision, Formal analysis, Data curation. **Masanori Kameoka:** Writing – review & editing, Validation, Supervision, Methodology. **Novi Anggraeni:** Investigation, Formal analysis. **Robby Nurhariansyah:** Project administration, Investigation. **Dominicus Husada:** Project administration, Investigation. **Citrawati Dyah Kencono Wungu:** Writing – review & editing, Visualization, Supervision, Software, Funding acquisition, Formal analysis, Conceptualization.

## Ethic approval and consent to participate

This study was approved by the Ethical Committees of Universitas Airlangga (Ethical Committee Approval Number: 123/KEH/2021). Written informed consent was obtained from all individual participants included in this study. All experiments were conducted in accordance to the relevant guidelines and regulations.

## Data availability statement

Data included in this article or data as supplementary material/references in the article are provided upon request.

## Additional information

No additional information is available for this paper.

## Funding statement

This work was supported by the program of the Japan Initiative for Global Research Network on Infectious Diseases (J-GRID) from the Ministry of Education, Culture, Sport, Science and Technology in Japan and the 10.13039/100009619Japan Agency for Medical Research and Development (10.13039/100009619AMED) and International Research Collaboration TOP 500 10.13039/501100008463Universitas Airlangga, Indonesia. (130/UN3.9.4/PT/2022).

## Declaration of competing interest

The authors declare that they have no known competing financial interests or personal relationships that could have appeared to influence the work reported in this paper.

## List of abbreviations

COVID-19: Coronavirus disease 2019
SARS-CoV-2: Severe acute respiratory syndrome coronavirus 2
NS1: nonstructural 1
RDT: rapid detection test
Abs: antibodies
ELISA: enzyme-linked immunosorbent assay
RT-PCR: real-time polymerase chain reaction
RBD: receptor binding domain
ACE2: angiotensin converting enzyme 2
Ig: immunoglobulin
SD: standard deviation

CIconfidence interval.
