# Supplementary material for: Cross-reactivity between dengue virus and SARS-CoV-2 antibodies: Confirmation study using specimens from dengue-infected patients before the COVID-19 pandemic
Source: Heliyon. 2024 Oct 18;10(21):e39099. doi: 10.1016/j.heliyon.2024.e39099 (PMC11550075; doi:10.1016/j.heliyon.2024.e39099)
Supplement: Multimedia component 2 [file mmc2.docx]

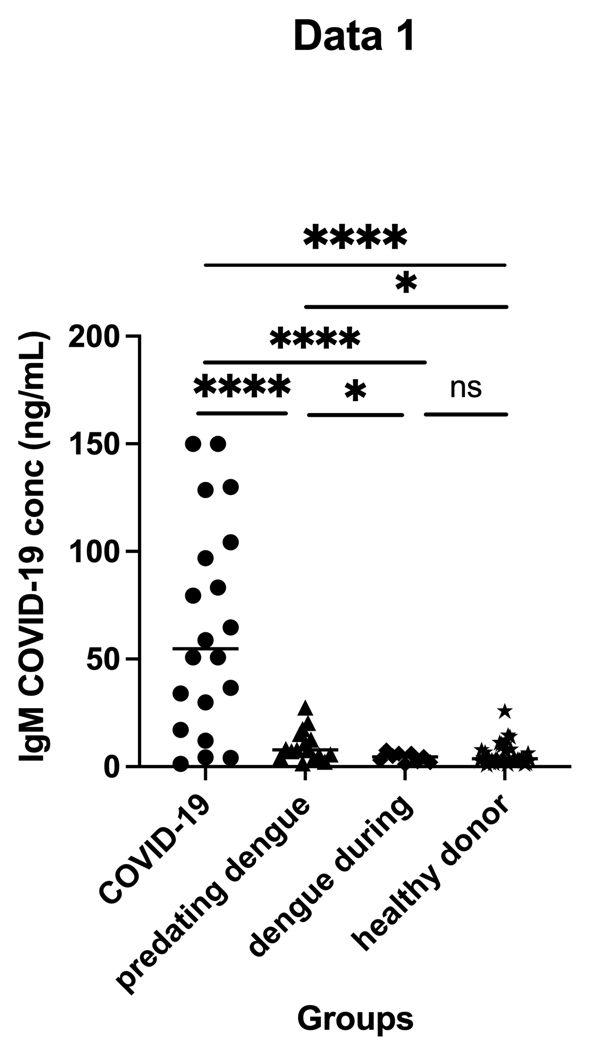

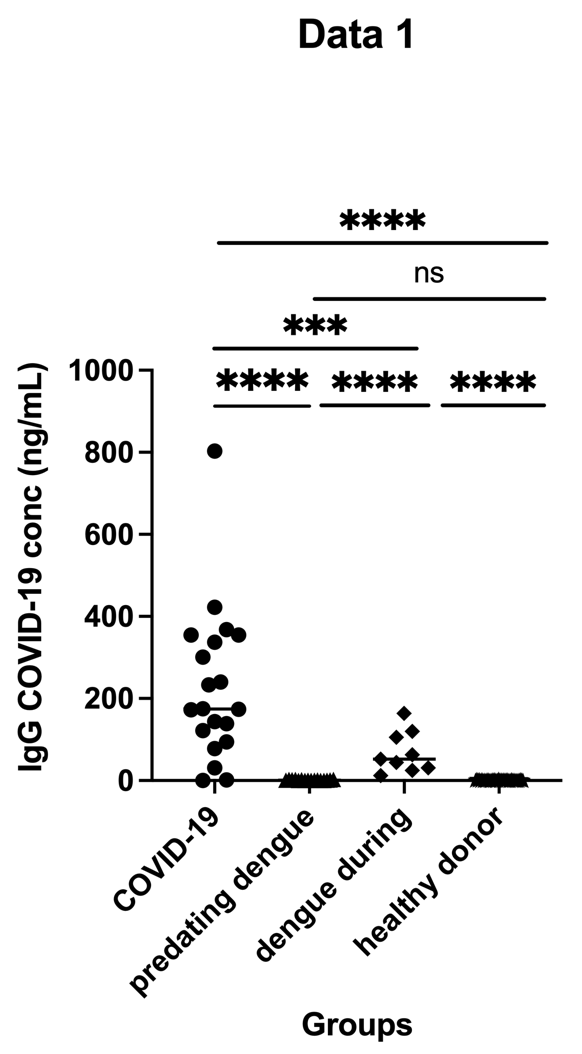


Supplementary figure 1. Analysis of (a) IgG and (b) IgM anti SARS-CoV2 ELISA between COVID-19, predating pandemic dengue, during pandemic dengue, and healthy donor samples. ns = non-significant; * = p < 0.05; *** = p < 0.01; **** = p < 0.0001
